# Supplementary material for: Resolving the full spectrum of human genome variation using Linked-Reads
Source: Genome Res. 2019 Apr;29(4):635–45. doi: 10.1101/gr.234443.118 (PMC6442396; doi:10.1101/gr.234443.118)
Supplement: Supplemental Material [file supp_gr.234443.118_Supplemental_longranger-2.2.zip › longranger-2.2.tar.bz2!/lr2.2-public/lib/stan/cmdstan/stan/doc/api/html/classstan_1_1math_1_1_l_d_l_t__factor_3_01stan_1_1agrad_1_1var_00_01_r_00_01_c_01_4.html]

Stan: stan::math::LDLT\_factor< stan::agrad::var, R, C > Class Template Reference


|  |  |
| --- | --- |
|  | Stan  2.5.0  probability, sampling & optimization |


- Main Page
- Related Pages
- Namespaces
- Classes
- Files

- Class List
- Class Index
- Class Hierarchy
- Class Members

All Classes Namespaces Files Functions Variables Typedefs Enumerations Enumerator Friends Macros Pages

- stan
- math
- LDLT\_factor< stan::agrad::var, R, C >

Public Types |
Public Member Functions |
Public Attributes |
List of all members

stan::math::LDLT\_factor< stan::agrad::var, R, C > Class Template Reference

A template specialization of src/stan/math/matrix/LDLT\_factor.hpp for stan::agrad::var which can be used with all the \*\_ldlt functions.
More...

`#include <LDLT_factor.hpp>`

|  |  |
| --- | --- |
| Public Types | |
| typedef size\_t | size\_type |
|  | |
| typedef stan::agrad::var | value\_type |
|  | |

|  |  |
| --- | --- |
| Public Member Functions | |
|  | LDLT\_factor () |
|  | Default constructor. More... |
|  | |
|  | LDLT\_factor (const Eigen::Matrix< stan::agrad::var, R, C > &A) |
|  | |
| void | compute (const Eigen::Matrix< stan::agrad::var, R, C > &A) |
|  | Use the LDLT\_factor object to factorize a new matrix. More... |
|  | |
| template<typename Rhs > | |
| const   Eigen::internal::solve\_retval  < Eigen::LDLT< Eigen::Matrix  < double, R, C > >, Rhs > | solve (const Eigen::MatrixBase< Rhs > &b) const |
|  | Compute the actual numerical result of inv(A)\*b. More... |
|  | |
| bool | success () const |
|  | Determine whether the most recent factorization succeeded. More... |
|  | |
| Eigen::VectorXd | vectorD () const |
|  | The entries of the diagonal matrix D. More... |
|  | |
| size\_t | rows () const |
|  | |
| size\_t | cols () const |
|  | |

|  |  |
| --- | --- |
| Public Attributes | |
| stan::agrad::LDLT\_alloc< R, C > \* | \_alloc |
|  | The LDLT\_alloc object actually contains the factorization but is derived from the chainable\_alloc class so that it is allocated on the vari stack. More... |
|  | |

## Detailed Description

### template<int R, int C> class stan::math::LDLT\_factor< stan::agrad::var, R, C >

A template specialization of src/stan/math/matrix/LDLT\_factor.hpp for stan::agrad::var which can be used with all the \*\_ldlt functions.

The usage pattern is:

Eigen::Matrix<T,R,C> A1, A2;

LDLT\_factor<T,R,C> ldlt\_A1(A1);

LDLT\_factor<T,R,C> ldlt\_A2;

ldlt\_A2.compute(A2);

Now, the caller should check that ldlt\_A1.success() and ldlt\_A2.success() are true or abort accordingly. Alternatively, call check\_ldlt\_factor(). The behaviour of using an LDLT\_factor without success() returning true is undefined.

Note that ldlt\_A1 and ldlt\_A2 are completely equivalent. They simply demonstrate two different ways to construct the factorization.

Now, the caller can use the LDLT\_factor objects as needed. For instance

x1 = mdivide\_left\_ldlt(ldlt\_A1,b1);

x2 = mdivide\_right\_ldlt(b2,ldlt\_A2);

d1 = log\_determinant\_ldlt(ldlt\_A1);

d2 = log\_determinant\_ldlt(ldlt\_A2);

Definition at line 45 of file LDLT\_factor.hpp.

## Member Typedef Documentation

template<int R, int C>

typedef size\_t stan::math::LDLT\_factor< stan::agrad::var, R, C >::size\_type

Definition at line 117 of file LDLT\_factor.hpp.

template<int R, int C>

typedef stan::agrad::var stan::math::LDLT\_factor< stan::agrad::var, R, C >::value\_type

Definition at line 118 of file LDLT\_factor.hpp.

## Constructor & Destructor Documentation

template<int R, int C>

|  |  |  |  |  |
| --- | --- | --- | --- | --- |
| stan::math::LDLT\_factor< stan::agrad::var, R, C >::LDLT\_factor | ( |  | ) |  |

inline

Default constructor.

The caller *MUST* call compute() after this. Any calls which use the LDLT\_factor without calling compute() run the risk of crashing Stan from within Eigen.

Definition at line 52 of file LDLT\_factor.hpp.

template<int R, int C>

|  |  |  |  |  |  |
| --- | --- | --- | --- | --- | --- |
| stan::math::LDLT\_factor< stan::agrad::var, R, C >::LDLT\_factor | ( | const Eigen::Matrix< stan::agrad::var, R, C > & | *A* | ) |  |

inline

Definition at line 54 of file LDLT\_factor.hpp.

## Member Function Documentation

template<int R, int C>

|  |  |  |  |  |
| --- | --- | --- | --- | --- |
| size\_t stan::math::LDLT\_factor< stan::agrad::var, R, C >::cols | ( |  | ) | const |

inline

Definition at line 115 of file LDLT\_factor.hpp.

template<int R, int C>

|  |  |  |  |  |  |
| --- | --- | --- | --- | --- | --- |
| void stan::math::LDLT\_factor< stan::agrad::var, R, C >::compute | ( | const Eigen::Matrix< stan::agrad::var, R, C > & | *A* | ) |  |

inline

Use the LDLT\_factor object to factorize a new matrix.

After calling this function, the user should call success() to check that the factorization was successful. If the factorization is not successful, the LDLT\_factor is not valid and other functions should not be used.

Parameters
:   |  |  |
    | --- | --- |
    | A | A symmetric positive definite matrix to factorize |

Definition at line 67 of file LDLT\_factor.hpp.

template<int R, int C>

|  |  |  |  |  |
| --- | --- | --- | --- | --- |
| size\_t stan::math::LDLT\_factor< stan::agrad::var, R, C >::rows | ( |  | ) | const |

inline

Definition at line 114 of file LDLT\_factor.hpp.

template<int R, int C>

template<typename Rhs >

|  |  |  |  |  |  |
| --- | --- | --- | --- | --- | --- |
| const Eigen::internal::solve\_retval<Eigen::LDLT< Eigen::Matrix<double,R,C> >, Rhs> stan::math::LDLT\_factor< stan::agrad::var, R, C >::solve | ( | const Eigen::MatrixBase< Rhs > & | *b* | ) | const |

inline

Compute the actual numerical result of inv(A)\*b.

Note that this isn't meant to handle any of the autodiff. This is a convenience function for the actual implementations in mdivide\_left\_ldlt.

Precondition: success() must return true. If success() returns false, this function runs the risk of crashing Stan from within Eigen.

Parameters
:   |  |  |
    | --- | --- |
    | b | The right handside. Note that this is templated such that Eigen's expression-templating magic can work properly here. |

Definition at line 85 of file LDLT\_factor.hpp.

template<int R, int C>

|  |  |  |  |  |
| --- | --- | --- | --- | --- |
| bool stan::math::LDLT\_factor< stan::agrad::var, R, C >::success | ( |  | ) | const |

inline

Determine whether the most recent factorization succeeded.

This should always be called after the object is constructed (with a matrix) or after compute() is called.

Definition at line 94 of file LDLT\_factor.hpp.

template<int R, int C>

|  |  |  |  |  |
| --- | --- | --- | --- | --- |
| Eigen::VectorXd stan::math::LDLT\_factor< stan::agrad::var, R, C >::vectorD | ( |  | ) | const |

inline

The entries of the diagonal matrix D.

They should be strictly positive for a positive definite matrix.

Precondition: success() must return true. If success() returns false, this function runs the risk of crashing Stan from within Eigen.

Definition at line 110 of file LDLT\_factor.hpp.

## Member Data Documentation

template<int R, int C>

stan::agrad::LDLT\_alloc<R,C>\* stan::math::LDLT\_factor< stan::agrad::var, R, C >::\_alloc

The LDLT\_alloc object actually contains the factorization but is derived from the chainable\_alloc class so that it is allocated on the vari stack.

This ensures that it's lifespan is longer than the LDLT\_factor object which created it. This is needed because the factorization is required during the chain() calls which happen after an LDLT\_factor object will most likely have been destroyed.

Definition at line 128 of file LDLT\_factor.hpp.

---

The documentation for this class was generated from the following file:

- src/stan/agrad/rev/matrix/LDLT\_factor.hpp


---

[ Stan Home Page ]

*© 2011–2014,
Stan Development Team.*
